# Metabolomic Profiling of Osteoblasts in Rat Subchondral Bone Following Anterior Cruciate Ligament Injury

**DOI:** 10.3390/molecules30112255

**Published:** 2025-05-22

**Authors:** Xu Qiu, Huili Deng, Xuchang Zhou, Guoxin Ni, Caihua Huang, Donghai Lin

**Affiliations:** 1 Key Laboratory for Chemical Biology of Fujian Province, High-Field NMR Research Center, College of Chemistry and Chemical Engineering, Xiamen University, Xiamen 361005, China; qiuxu@stu.xmu.edu.cn; 2 Department of Rehabilitation Medicine, The First Affiliated Hospital of Xiamen University, School of Medicine, Xiamen University, Xiamen 361005, China; 19373142312@163.com (H.D.); mdzxsj@163.com (X.Z.); 3 Research and Communication Center of Exercise and Health, Xiamen University of Technology, Xiamen 361024, China; huangcaihua@xmut.edu.cn

**Keywords:** anterior cruciate ligament injury, post-traumatic osteoarthritis, osteoblasts, metabolomic profiling

## Abstract

**Objectives:** Osteoarthritis (OA) is a prevalent chronic degenerative joint disorder marked by cartilage degradation, subchondral bone remodeling, and synovial inflammation. Despite its widespread occurrence, effective pharmacological interventions to halt or reverse OA progression remain elusive. Thus, an in-depth understanding of its pathogenesis is imperative for developing novel therapeutic strategies. **Methods:** Sixty-four male Sprague-Dawley rats (8 weeks old, weighing 180–220 g) were randomly assigned to two groups: the anterior cruciate ligament transection (ACLT) group and the sham-operated group. Primary osteoblasts were isolated from the subchondral bone at 0, 4, 8, and 12 weeks after ACLT. Nuclear magnetic resonance (NMR)-based metabolomics was used to elucidate metabolic changes and the underlying mechanisms in osteoblasts. **Results:** A total of 26 metabolites were identified from the NMR spectra of osteoblasts. Distinct metabolic profiles were observed in the ACLT group at 0, 4, 8, and 12 weeks after surgery. In particular, several differential metabolites were identified, including glucose, lactate, NADP^+^, phosphocreatine, and alanine, as well as eight perturbed pathways, such as alanine, aspartate, and glutamate metabolism, phenylalanine metabolism, and taurine metabolism. **Conclusions:** Key energy-related metabolites, including glucose, lactate, creatine phosphate, and creatine, were identified as key markers of osteoblast dysfunction in OA, underscoring the profound metabolic perturbations induced by ACL injury. These disturbances in energy homeostasis are strongly implicated in the progression of OA. In addition, branched-chain amino acids emerged as potential biomarkers, further highlighting the metabolic dysregulation associated with the disease. Taken together, the metabolic changes observed in rat osteoblasts following ACL injury reveal a complex interplay between energy and amino acid metabolism, providing critical insights into the pathogenesis of post-traumatic OA and highlighting potential therapeutic targets.

## 1. Introduction

Osteoarthritis (OA) is a chronic degenerative disease characterized by progressive cartilage degradation, abnormal subchondral bone remodeling, osteophyte formation, and persistent synovial inflammation. It is the most common form of arthritis and a leading cause of disability worldwide [1]. The growing burden of OA is exacerbated by an aging population and increasing obesity rates, with risk factors including age, joint injury, obesity, genetic predisposition, anatomical variations, and gender [2].

Post-traumatic osteoarthritis (PTOA) occurs after joint injury and accounts for approximately 12% of symptomatic OA cases [3]. Anterior cruciate ligament (ACL) injuries, meniscal tears, and glenohumeral instability significantly contribute to the development of PTOA, with ACL injuries alone predisposing individuals to an 87% incidence of PTOA [4,5]. The ACL transection (ACLT) model has been extensively validated as a translational platform for PTOA research, demonstrating >85% concordance with human disease progression in key pathologic features when monitored beyond 8 weeks post-injury [6]. Through biomechanical destabilization, this approach reliably reproduces the triphasic progression of human PTOA: acute synovitis (0–4 weeks), subchondral bone remodeling (4–8 weeks), and terminal cartilage degradation (8–12 weeks) [7]. Systematic analyses confirm its ability to mimic clinical biomarkers, including sustained elevation of serum COMP (2.5–3.2 fold) and CTX-II (3.0–4.1 fold) levels [8], while micro-CT quantification reveals characteristic subchondral sclerosis (bone volume/total volume increase ≥15%) [9].

While spontaneous OA and PTOA diverge in initiating mechanisms (age-related degeneration vs. acute trauma), they converge on common terminal pathways involving metabolic dysregulation (e.g., aberrant glycolysis, oxidative stress) and osteoblast dysfunction [10,11]. For instance, ACLT recapitulates early metabolic signatures of OA, including lactate accumulation and anaerobic glycolysis, which are consistent with metabolomic profiles observed in synovial fluid from human OA patients [11,12]. Mechanistically, both models exhibit sustained biomechanical stress and pro-inflammatory cytokine cascades (e.g., IL-1β, TNF-α) that drive osteoblast metabolic reprogramming [7,13]. This congruence underscores the utility of ACLT for investigating injury-induced metabolic perturbations relevant to OA pathogenesis. Crucially, longitudinal metabolomic studies confirm that ACLT-derived osteoblasts reflect metabolic shifts (e.g., BCAA accumulation, NADP^+^ depletion) that were later validated in human OA tissues [11,12], solidifying its relevance for investigating OA-associated metabolic derangements.

Although transcriptional signatures of osteoblasts differ between ACLT and natural OA [14], these disparities primarily reflect divergent initiating triggers rather than terminal metabolic outcomes. Emerging evidence underscores the critical role of periarticular structures, particularly the subchondral bone, in disease progression. Subchondral bone thickening is a hallmark of OA [15], with ACL injuries often triggering an imbalance between bone resorption and formation, leading to structural deterioration [5,16,17]. Advanced imaging studies have revealed microstructural changes in subchondral bone, including early bone loss and subsequent sclerosis, indicating complex interactions within the osteochondral unit [18,19].

Despite the availability of symptom-relief therapies, no treatment currently halts the progression of OA, underscoring the urgent need for early diagnostic markers and targeted interventions [20]. A comprehensive characterization of mechanobiological and metabolic alterations in subchondral bone provides critical insights into the fundamental mechanisms underlying OA pathogenesis.

Metabolomics, particularly nuclear magnetic resonance (NMR) spectroscopy, has emerged as a powerful tool for identifying diagnostic and prognostic biomarkers by profiling metabolite changes [21,22]. The non-invasive nature and quantitative precision of NMR make it an invaluable technique for elucidating metabolic perturbations in OA [23,24]. Recent ACLT-based metabolomic studies have successfully identified disease-associated pathways (e.g., dysregulation of purine metabolism) that were later validated in human synovial fluid [11], establishing this model as a discovery platform with direct clinical relevance. Although previous metabolomics studies have examined synovial fluid and subchondral bone metabolites across various models, there remains a critical gap in understanding the metabolic characteristics of osteoblasts in OA [25,26,27,28].

The precisely timed injury onset in PTOA creates an advantageous experimental window for probing initial pathogenic cascades. Current limitations in delineating disease-driving mechanisms [29,30,31] motivated our use of longitudinal NMR metabolomics to track ACLT-induced metabolic adaptations in rat osteoblasts. By constructing temporal metabolic networks, this work reveals preliminary evidence of pathway dysregulations coinciding with structural remodeling. These data-driven observations provide hypothesis-generating insights into the metabolic dimension of PTOA progression.

## 2. Results

### 2.1. Histological Changes in Bone Tissue Following Anterior Cruciate Ligament Transection (ACLT)

**Figure 1:**
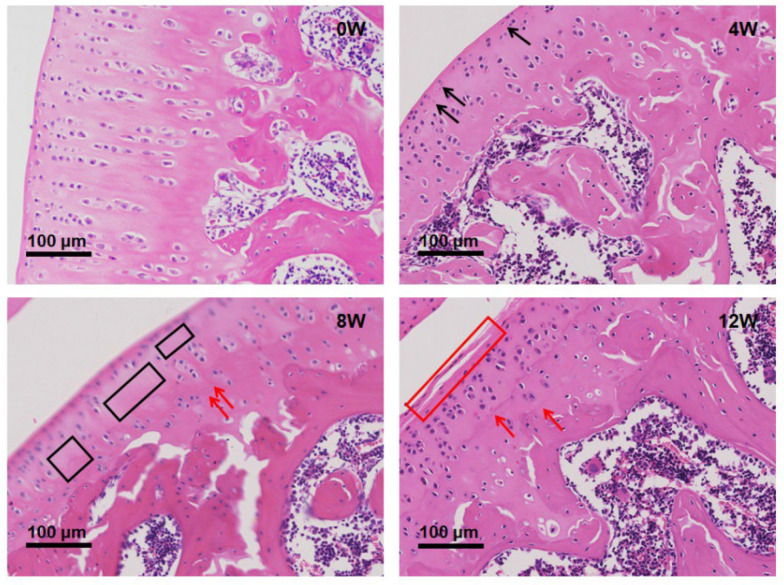
Histological changes in subchondral bone at different time points after ACLT in rats. Representative H&E-stained sections illustrate the progressive structural deterioration of subchondral bone at 0, 4, 8, and 12 weeks post-ACLT. At 0 weeks (baseline), the cartilage structure remains intact with well-organized subchondral trabecular bone. At 4 weeks, early pathological changes appear, including cartilage erosion, trabecular bone loss, and inflammatory cell infiltration, and chondrocyte death and disordered arrangement begin to appear (black arrow). By 8 weeks, increased bone resorption and fibrotic tissue deposition are evident, the number of chondrocytes in some cartilage areas was significantly reduced (black box), and there was tidemark replication (red arrow). At 12 weeks, severe cartilage degradation, extensive trabecular bone rarefaction and marked osteoarthritic changes are observed, fibrosis and partial detachment occurred in the superficial layer of cartilage (red box), and there was significant tidemark replication (red arrow). Scale bars: 100 μm.

Hematoxylin and Eosin (H&E) staining was performed on bone tissue at 0, 4, 8 and 12 weeks post-ACLT to assess structural changes associated with osteoarthritis (OA) progression (Figure 1).

At 0 weeks (baseline), the cartilage structure was intact, with a smooth articular surface and dense subchondral trabecular bone, showing no signs of degeneration or bone loss.

At 4 weeks, early pathological changes appeared, including cartilage surface erosion and thinning, accompanied by subchondral trabecular bone loss, expansion of the marrow cavity and infiltration of inflammatory cells. These changes suggest an initial inflammatory response after ACLT.

At 8 weeks, the degeneration became more pronounced, with progressive cartilage degradation, a significant decrease in subchondral trabecular bone, and increased bone resorption. In addition, fibrotic tissue deposition was observed, indicating an attempt at structural remodeling.

At 12 weeks, severe osteoarthritic changes were evident, characterized by extensive cartilage loss, destruction of the subchondral bone architecture, and marked trabecular rarefaction. The significant bone loss and osteoporotic features at this stage reflect advanced joint degeneration.

These results suggest that ACLT induces a progressive decline in bone integrity, mirroring the pathological features of OA and highlighting the critical role of subchondral bone remodeling in disease progression.

### 2.2. Morphological Observation and Alkaline Phosphatase (ALP) Staining Results of Primary Osteoblasts

The results of primary osteoblast identification through microscopic observation and ALP staining are presented in Figure S1. Microscopic examination (Figure S1A) revealed that the primary osteoblasts had typical morphological characteristics. The cells had a spindle-shaped or polygonal appearance with a relatively uniform distribution and showed good adhesion to the culture surface, which is consistent with the morphological features of osteoblasts reported in previous studies [32,33]. This morphological observation is a first indication of the successful isolation of primary osteoblasts.

For the ALP staining results (Figure S1B), a significant amount of blue-violet precipitate was observed within the cells, indicating a high level of ALP activity. ALP is a key marker for osteoblasts, and the presence of such staining confirms the osteogenic nature of the isolated cells [33]. The extensive and intense staining suggests that the primary osteoblasts are actively expressing ALP, further validating that the cells isolated and cultured in this study are indeed osteoblasts.

These results provide strong evidence for the subsequent metabolomics experiments on primary osteoblasts, laying a solid foundation for exploring the metabolic profiles and regulatory mechanisms of osteoblasts.

### 2.3. Metabolite Identification in Osteoblasts

**Figure 2:**
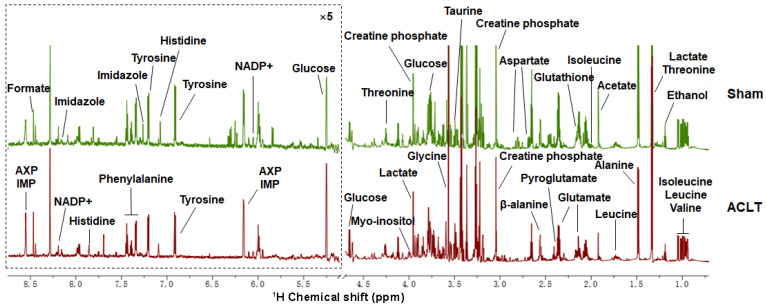
Representative 850 MHz 1D ^1^H-NMR spectra of aqueous metabolites extracted from osteoblasts in the sham and ACLT groups at pH 7.4 and 298 K. The vertical scale was uniformly maintained for both spectra. The displayed region spans from 0.50 to 8.75 ppm, excluding the water resonance region (4.70–4.85 ppm). To enhance clarity, the resonance region of 4.85–8.75 ppm has been magnified fivefold relative to that of 0.50–4.70 ppm. Key metabolite peaks are labeled, highlighting distinct metabolic differences between groups.

A total of 26 aqueous metabolites were identified in osteoblasts using one-dimensional (1D) ^1^H-NMR spectroscopy (Figure 2). Resonance assignments were further validated through two-dimensional (2D) ^1^H-^1^H total correlation spectroscopy (TOCSY) and ^1^H-^13^C heteronuclear single quantum coherence (HSQC) spectra, ensuring high confidence in metabolite identification (Figures S2 and S3). A comprehensive list of resonance assignments is provided in Supplementary Table S1, offering detailed insights into the metabolic profile of osteoblasts following anterior cruciate ligament (ACL) injury.

These findings highlight the intricate metabolic landscape of osteoblasts, establishing a foundation for subsequent analyses aimed at elucidating the role of these metabolites in osteoblast function and their contribution to OA pathogenesis.

### 2.4. Metabolic Profiles of Osteoblasts at Different Time Points After ACLT

**Figure 3:**
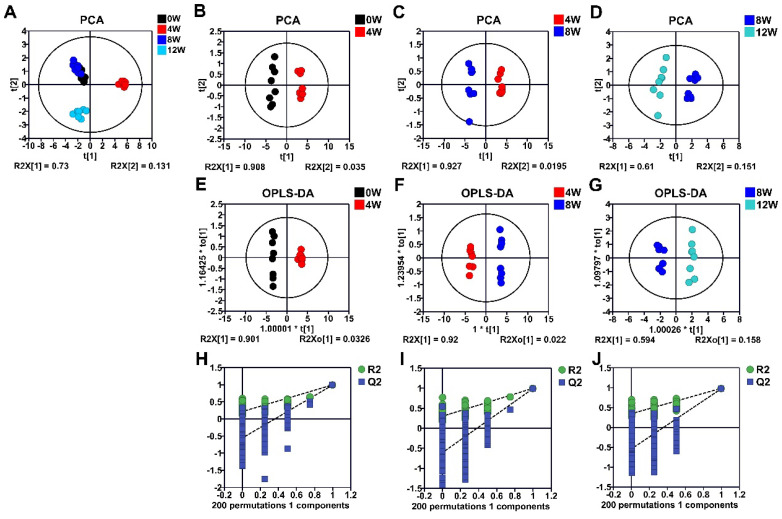
Multivariate statistical analysis of the NMR dataset to reveal metabolic differences in osteoblasts at different time points after ACLT. (**A**) Overall PCA score plot representing all four groups. (**B**–**D**) Detailed PCA score plots highlighting distinctions between specific pairs: 4 W vs. 0 W (**B**), 8 W vs. 4 W (**C**), and 12 W vs. 8 W (**D**). (**E**–**G**) OPLS-DA score plots illustrating the metabolic differentiation between the 4 W and 0 W groups (**E**), the 8 W and 4 W groups (**F**), and the 12 W and 8 W groups (**G**). (**H**–**J**) Cross-validation plots for assessing the robustness of the OPLS-DA models, corresponding to panels (**E**–**G**), respectively.

Principal component analysis (PCA) and orthogonal partial least squares discriminant analysis (OPLS-DA) score plots revealed significant metabolic variations at different time points following ACLT (Figure 3). Pairwise comparisons between adjacent time points yielded the following model performance parameters: 4 weeks (4 W) vs. 0 weeks (0 W, baseline), R^2^_Y_(cum) = 0.995, Q^2^_Y_(cum) = 0.992 (Figure 3B); 8 weeks (8 W) vs. 4 weeks (4 W), R^2^_Y_(cum) = 0.997, Q^2^_Y_(cum) = 0.991 (Figure 3E); 12 weeks (12 W) vs. 8 weeks (8 W), R^2^_Y_(cum) = 0.986, Q^2^_Y_(cum) = 0.975 (Figure 3H).

Here, R^2^_Y_ (cum) represents the goodness of fit, indicating how well the model explains the variance, while Q^2^_Y_ (cum) reflects the predictive accuracy of the OPLS-DA model. The consistently high R^2^_Y_ (cum) and Q^2^_Y_ (cum) values confirm the reliability and robustness of the established models.

Furthermore, it was observed that the distribution trends of samples at different time points subsequent to ACLT on the PCA plot were of considerable interest (Figure 3A). Specifically, the metabolic patterns of samples in the 0 W, 4 W, and 12 W groups following ACLT were distinctly separated. In comparison to the substantial metabolic disparities between the 4 W and 12 W groups, the metabolic profile of the 8 W group appeared to bear a closer resemblance to that of the 0 W group.

To delve deeper into the metabolic differences between the 8 W and 0 W time points post-ACLT, a further analysis of the 8 W vs. 0 W comparison was carried out (Figure S4). The findings indicated that the metabolic patterns of the 8 W and 0 W groups after ACLT were also markedly different. Specifically, the values of R^2^_Y_ (cum) and Q^2^_Y_ (cum) for the OPLS-DA model were 0.979 and 0.953, respectively.

Collectively, these analyses reveal significant metabolic changes in osteoblasts following ACLT, underscoring the dynamic metabolic shifts that drive OA progression.

### 2.5. Differencial Metabolites in Osteoblasts at Different Time Points After ACLT

One-way ANOVA analysis revealed significant metabolic changes at different time points post-ACLT (Table 1) and identified differential metabolites with significant concentration changes (FDR-adjusted *p* < 0.05): 4 W vs. 0 W (baseline), 21 differential metabolites; 8 W vs. 4 W, 22 differential metabolites, 12 W vs. 8 W, 22 differential metabolites.

These results demonstrate a dynamic metabolic response over time, highlighting progressive changes in osteoblast metabolism after ACLT. The consistent appearance of differential metabolites across multiple time points highlights their potential role in OA pathogenesis and disease progression.

### 2.6. Significant Metabolites in Osteoblasts at Different Time Points After ACLT

**Figure 4:**
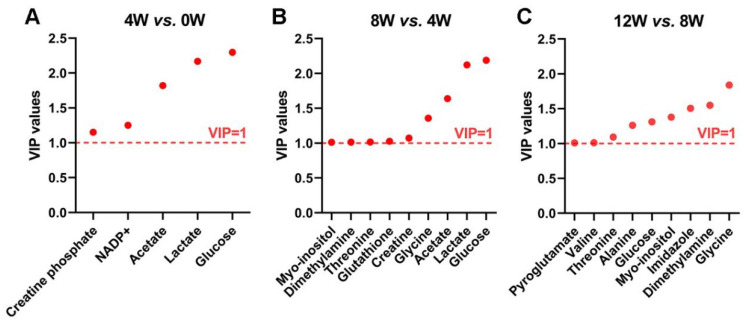
Identification of significant metabolites from pairwise comparisons of osteoblasts at different time points after ACLT. VIP score-ranking plots from the OPLS-DA model highlight significant metabolites contributing to metabolic separation between groups: (**A**) 4 W vs. 0 W, (**B**) 8 W vs. 4 W, and (**C**) 12 W vs. 8 W. The red dashed line represents the threshold (VIP = 1), where metabolites above this cut-off are considered significant in differentiating metabolic profiles.

Significant metabolites were identified using the OPLS-DA model with a variable importance in projection (VIP) score > 1 (Figure 4). Comparative analysis of metabolite profiles at adjacent time points post-ACLT revealed distinct metabolic shifts: 4 W vs. 0 W, five significant metabolites; 8 W vs. 4 W, nine significant metabolites; 12 W vs. 8 W, nine significant metabolites.

These results illustrate a dynamic metabolic landscape in osteoblasts during the recovery phase, with notable metabolic shifts occurring particularly between 4 and 8 weeks post-ACLT. This period may represent a critical window for metabolic adaptation, highlighting potential biomarkers and therapeutic targets for OA progression.

### 2.7. Characteristic Metabolites of Osteoblasts at Different Time Points After ACLT

**Figure 5:**
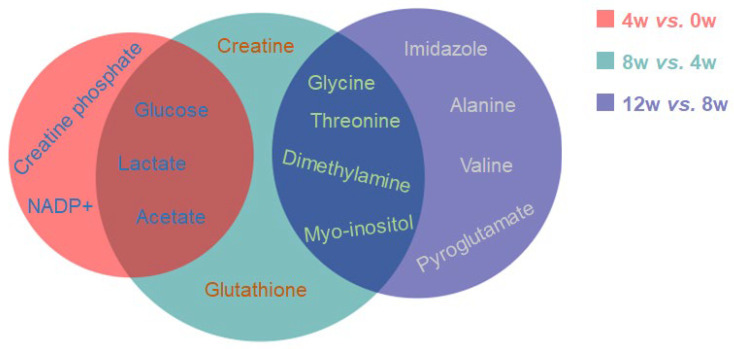
Venn diagram of characteristic metabolites identified from pairwise comparisons of osteoblasts at different time points after ACLT. The diagram illustrates the overlap and distinct characteristic metabolites identified in 4 W vs. 0 W (red), 8 W vs. 4 W (green), and 12 W vs. 8 W (purple).

Characteristic metabolites were identified using the criteria variable importance in projection (VIP) > 1 and adjusted *p* < 0.05, as summarized in Table 2. The analysis revealed distinct metabolic shifts at different time points after ACLT: 4 W vs. 0 W: five characteristic metabolites, 8 W vs. 4 W, nine characteristic metabolites; 12 W vs. 8 W, eight characteristic metabolites. The Venn diagram (Figure 5) illustrates the overlap of characteristic metabolites across time points. Notably, three common characteristic metabolites—glucose, lactate, and acetate—were shared between the 4 W vs. 0 W and 8 W vs. 4 W comparisons, suggesting their involvement in early metabolic adaptations after ACLT. Similarly, glycine, threonine, dimethylamine, and myo-inositol were identified as common characteristic metabolites between 8 W vs. 4 W and 12 W vs. 8 W, suggesting their potential role in later metabolic transitions associated with osteoblast function and OA progression.

**Figure 6:**
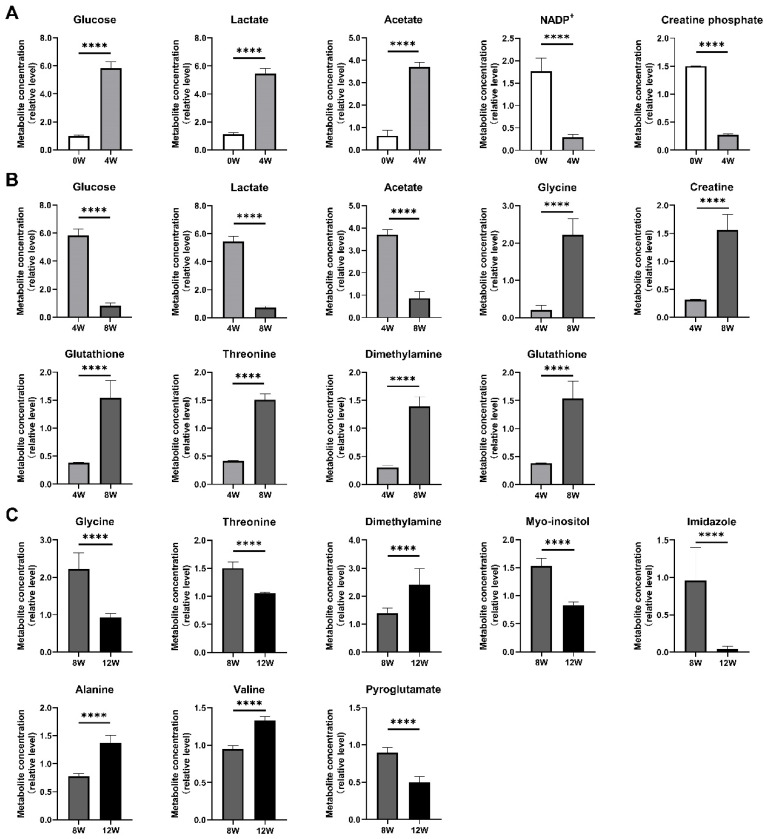
Temporal profiles of characteristic metabolite concentrations in osteoblasts at different time points after ACLT. Bar graphs show the relative concentration changes of key metabolites in three pairwise comparisons: (**A**) 4 W vs. 0 W, (**B**) 8 W vs. 4 W, and (**C**) 12 W vs. 8 W. Metabolite levels were quantified based on NMR spectral integration and normalized for comparative analysis. Statistical significance was determined by one-way ANOVA, with significance levels indicated as follows: **** adjusted *p* < 0.0001. These findings highlight dynamic metabolic changes in osteoblasts during OA progression, particularly in pathways related to energy metabolism, amino acid metabolism, and oxidative stress regulation.

These results demonstrate a dynamic metabolic profile in osteoblasts, characterized by significant fluctuations at each time point (Figure 6). Such changes may play a key role in the metabolic dysregulation that contributes to the progression of OA.

### 2.8. Key Metabolic Pathways in Osteoblast at Different Time Points Following ACLT

**Figure 7:**
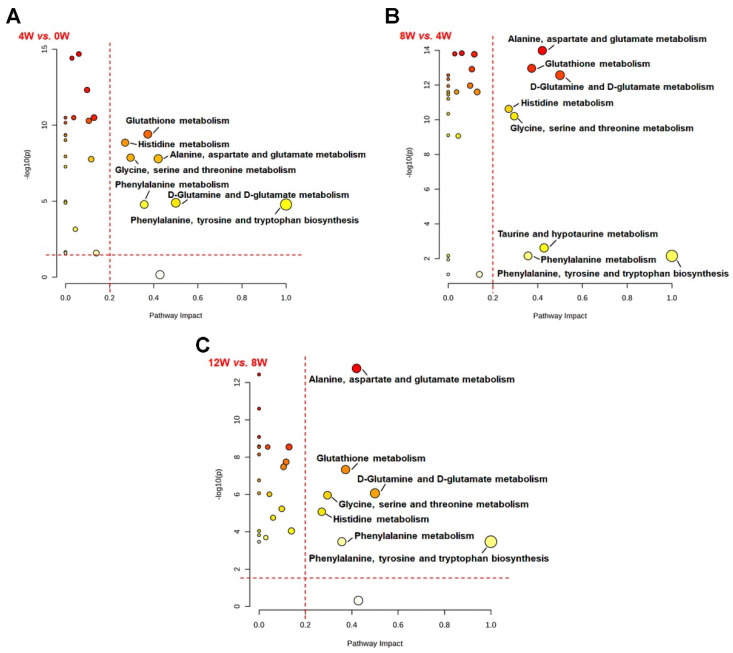
Metabolic pathway analysis of osteoblasts at different time points after ACLT. Significantly altered pathways were identified from pairwise comparisons using the criteria of PIV > 0.2 and *p* < 0.05: (**A**) 4 W vs. 0 W, (**B**) 8 W vs. 4 W, and (**C**) 12 W vs. 8 W. The *x*-axis represents pathway impact, while the *y*-axis indicates statistical significance (−log_10_(*p*)). Larger and darker colored circles indicate pathways with greater impact and statistical significance.

The identified significantly altered metabolic pathways over the time course after ACLT are shown in Figure 7, including the following: 4 W vs. 0 W, seven significantly altered pathways; 8 W vs. 4 W, eight significantly altered pathways; 12 W vs. 8 W, seven significantly altered pathways (Table 3).

Of the key metabolic pathways identified, the following five pathways were significantly disturbed: glutathione metabolism; histidine metabolism; alanine, aspartate, and glutamate metabolism; glycine, serine, and threonine metabolism; and phenylalanine metabolism. In addition, three pathways related to D-glutamine and D-glutamate metabolism, phenylalanine, tyrosine, and tryptophan biosynthesis, and taurine and hypotaurine metabolism were closely associated with osteoblast activation in osteoarthritic subchondral bone.

**Figure 8:**
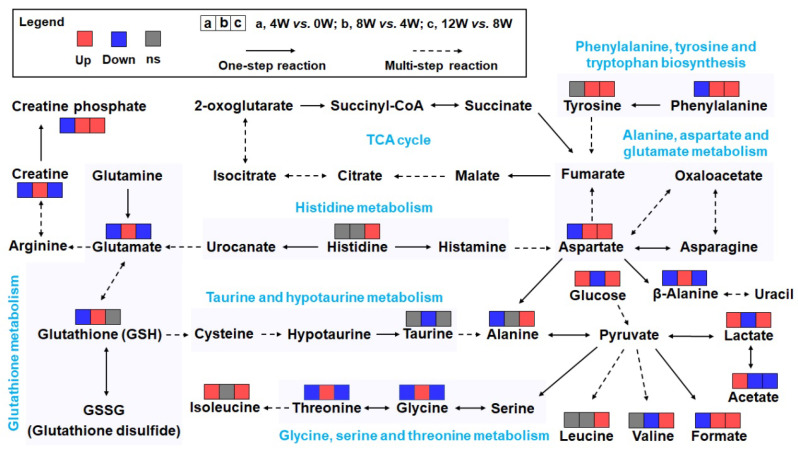
Schematic representation of metabolic changes in significantly affected pathways in osteoblasts at different time points after ACLT. Dotted arrows indicate multi-step reactions, whereas solid arrows represent single-step reactions. Changes in metabolite concentrations are color coded: red indicates an increase, blue indicates a decrease, and gray indicates no significant change. The schematic highlights key pathways, including glutathione metabolism; alanine, aspartate, and glutamate metabolism; glycine, serine, and threonine metabolism; taurine and hypotaurine metabolism; and phenylalanine, tyrosine, and tryptophan biosynthesis, illustrating the complex metabolic network affected by ACL injury and OA progression.

To further elucidate the relationships between characteristic metabolites and significantly altered pathways, we constructed a schematic diagram based on the KEGG data (Figure 8). This visualization provides a comprehensive overview of key metabolic interactions and highlights the intricate metabolic network underlying OA progression. These findings highlight the critical role of osteoblasts in responding to metabolic perturbations following ACLT and provide valuable insights into the pathophysiology of OA.

## 3. Discussion

Subchondral bone remodeling, controlled by the dynamic interplay between osteoblasts and osteoclasts, is essential for maintaining bone homeostasis. Disruption of this balance is recognized as an early contributor to the progression of osteoarthritis (OA). In this study, we used an NMR-based metabolomics approach to characterize dynamic metabolic shifts in osteoblasts following anterior cruciate ligament transection (ACLT) in a rat model. Our results provide compelling evidence that metabolic dysregulation within the subchondral bone plays a pivotal role in the pathogenesis of OA.

Consistent with previous studies [34,35,36], we observed that metabolic alterations in osteoblasts occur before detectable cartilage degeneration, suggesting that these early biochemical perturbations may serve as a trigger for OA development (Figure S5). Multivariate statistical analysis revealed significant metabolic changes at 4, 8, and 12 weeks post-ACLT, with a pronounced impact on energy metabolism pathways. In particular, fluctuations in key metabolites including glucose, lactate, creatine phosphate, and branched-chain amino acids (BCAAs) suggest a progressive shift in osteoblast metabolic activity. These findings underscore the profound biochemical consequences of anterior cruciate ligament (ACL) injury and implicate osteoblast dysfunction as a driving force behind OA, which is associated with subchondral bone changes.

Notably, while ACL injury-induced post-traumatic osteoarthritis (PTOA) and degenerative OA differ in their initiating mechanisms (acute trauma vs. age-related degeneration), both converge on common terminal pathways involving osteoblast metabolic reprogramming [10,11]. For instance, the observed BCAA accumulation and NADP^+^ depletion in ACLT-derived osteoblasts matches the metabolomic profiles from human OA synovial fluid [12], supporting the translational relevance of our model. This mechanistic overlap suggests that osteoblast dysfunction, whether triggered by mechanical destabilization or chronic degeneration, may disrupt energy homeostasis through similar pathways, such as mTORC1 activation and redox imbalance [37,38].

### 3.1. Metabolic Changes in Energy Substrates

Our analysis identified 26 different metabolites, with notable fluctuations in key energy-related metabolites, including glucose, lactate, formate, acetate, creatine, creatine phosphate, and NADP^+^. At 4 weeks post-ACLT, glucose levels were significantly elevated, consistent with the findings of Mickiewicz et al. [39], who reported similar metabolic shifts in synovial fluid. This glucose accumulation may be due to increased cytokine activity and matrix metalloproteinase (MMP) production, reflecting the increased energy demands associated with early joint repair [13,40].

In addition, the elevated lactate levels suggest a metabolic shift towards anaerobic glycolysis, likely driven by cellular stress and localized hypoxia within the injured joint. While this glycolytic adaptation may temporarily sustain energy production, excessive lactate accumulation could exacerbate local inflammation and tissue damage, a phenomenon commonly observed in the progression of OA [41].

The observed decrease in creatine, creatine phosphate, and NADP^+^ levels further suggests that osteoblasts may experience metabolic exhaustion as they attempt to meet increased energy demands. A reduction in NADP^+^ may also compromise redox homeostasis, impairing the ability of osteoblasts to counteract oxidative stress and sustain anabolic processes such as collagen synthesis [37,42]. These findings highlight the critical role of energy metabolism in osteoblast function and suggest that targeting metabolic pathways may represent novel therapeutic strategies to mitigate OA progression.

Interestingly, the Venn diagram revealed no overlapping metabolites across all three time points, underscoring the temporal specificity of metabolic adaptations. For example, glucose and lactate dominated the acute phase (4 W), while BCAAs and alanine emerged as key players in later stages (8 W–12 W). This dynamic shift likely reflects the phased progression of PTOA—acute inflammation, subchondral remodeling, and terminal cartilage degradation—each demanding distinct metabolic adaptations [7]. The absence of common metabolites highlights the complexity of OA pathogenesis and suggests that therapeutic strategies may need to target time-specific metabolic nodes.

### 3.2. Role of BCAA Metabolism

This study also examined the temporal dynamics of BCAA metabolism, focusing on leucine, isoleucine, and valine, which serve as essential substrates for energy metabolism and have been implicated in the pathogenesis of OA [43,44,45]. A significant upregulation of BCAA levels at 12 weeks post-ACLT suggests either impaired BCAA catabolism or increased proteolytic release in response to injury. These findings are consistent with Damyanovich et al. [12], who reported that elevated BCAA levels contribute to the pro-inflammatory environment characteristic of OA.

The involvement of BCAAs in mTORC1 signaling activation further supports their role in exacerbating inflammation and accelerating joint tissue degradation [12,38]. Elevated BCAA levels have been associated with increased expression of pro-inflammatory cytokines, including IL-1, IL-2, TNF-α, and IFN-γ, which are known to promote cartilage matrix degradation [46].

This dual role of BCAAs—acting as essential components for tissue repair while also amplifying inflammation—underscores the importance of regulating BCAA metabolism during OA progression [47,48].

In light of these findings, targeting BCAA-related pathways may represent a novel therapeutic strategy to alleviate OA-associated inflammation and preserve joint integrity. To mitigate BCAA-driven inflammation in early OA, targeted interventions such as mTORC1 inhibitors (e.g., rapamycin) or dietary modulation of BCAA intake could be explored [37,38]. Additionally, enhancing BCAA catabolism via activators of branched-chain ketoacid dehydrogenase (BCKDH) might restore metabolic equilibrium [12]. Early-phase clinical studies in OA patients could validate these approaches, particularly in populations with traumatic joint injuries.

### 3.3. Alanine Metabolism and Osteoblast Function

Our analysis revealed significant changes in alanine metabolism throughout the post-ACLT period. Alanine, a key amino acid involved in the alanine-glucose and tricarboxylic acid (TCA) cycle, plays a critical role in energy supply and muscle function [49,50]. We observed a marked decrease in alanine levels at 8 weeks post-ACLT, followed by a significant increase at 12 weeks, suggesting a biphasic metabolic response. This fluctuation may reflect the changing energy requirements of osteoblasts as they transition from early inflammatory responses to later stages of tissue repair and remodeling [51,52].

Interestingly, elevated alanine levels have been correlated with subchondral bone sclerosis, further supporting the hypothesis that osteoblast metabolic activity is closely linked to increased energy expenditure following ACL injury [53]. In light of these findings, targeting alanine metabolism may provide a therapeutic opportunity to modulate osteoblast function and slow the progression of OA. In particular, regulation of the alanine-glucose cycle may not only improve osteoblast energy efficiency but also provide insight into broader systemic metabolic shifts associated with OA pathogenesis [50,53].

### 3.4. Limitations

Despite the meaningful insights gained in this study, several limitations warrant acknowledgment. Although the ACLT model effectively recapitulates critical features of human PTOA, it may not fully capture the persistent low-grade inflammation characteristic of spontaneous OA. Additionally, the complex metabolic interplay among osteoblasts, chondrocytes, and synovial cells remains to be thoroughly elucidated and requires further investigation.

## 4. Materials and Methods

### 4.1. Osteoarthritis (OA) Animal Models

This study was approved by the Experimental Animal Ethics Committee of Fujian Medical University and conducted in accordance with institutional guidelines (Approval No. 005 [2018]). Rats were obtained from the Animal Experiment Center of Fujian Medical University. They were housed under controlled conditions with a temperature of 23–25 °C and a relative humidity of 40–60%. Artificial lighting was used to maintain a 12 h light/dark cycle. The rats were provided with standard chow and had free access to food and water.

Sixty-four male Sprague-Dawley rats (8 weeks old, 180–220 g) were randomly assigned to two groups: the anterior cruciate ligament transection (ACLT) group (*n* = 32) and the sham-operated control group (*n* = 32). The sham group, which underwent identical surgical procedures (medial parapatellar incision and joint exposure) except for anterior cruciate ligament (ACL) transection, served a dual purpose in this study. It served as an important control to distinguish the specific metabolic effects of ACL injury from general surgical trauma, to control for non-specific surgical effects (such as inflammation due to joint exposure), and to provide a baseline for normalization of metabolite levels to eliminate the effects of age- or time-related changes. To enhance data reliability and reduce biological variability, each group included at least eight rats for metabolomic analysis. Each group was further subdivided based on euthanasia time points (0, 4, 8, and 12 weeks post-surgery, *n* = 8 per subgroup).

OA was induced via ACL transection under isoflurane anesthesia. A medial parapatellar incision was made to expose the knee joint, followed by careful transection of the ACL under a surgical microscope to prevent cartilage damage. The joint capsule and patella were repositioned, and the wound was sutured in layers.

Postoperative management included daily intramuscular administration of penicillin (400,000 units) for three days to prevent infection. No immobilization measures were applied, allowing natural joint loading during recovery.

### 4.2. Hematoxylin and Eosin (H&E) Staining Procedure

Rat knee samples were decalcified in 10% ethylenediaminetetraacetic acid (EDTA) solution for six weeks, with the solution changed every two days to ensure uniform and consistent decalcification. Decalcification was considered complete when the bone tissue could be easily penetrated with a syringe.

After decalcification, samples were dehydrated through a graded ethanol series (50%, 70%, 85%, 95%, and 100%), with each concentration applied for 1–2 h to remove residual moisture. The specimens were then cleared in xylene for 15–30 min to make them transparent for optimal paraffin infiltration.

For paraffin embedding, samples were immersed in molten paraffin (58–62 °C) for 2–3 h to ensure complete infiltration. After embedding, 4 μm thick sections were cut on a microtome. Sections were deparaffinized in xylene (two cycles, 10–15 min each) and rehydrated through a descending ethanol series (100%, 95%, 85%, 70%, and 50%), with each step lasting 5–10 min.

Standard H&E staining was performed, resulting in blue-violet stained nuclei and pink-stained cytoplasm. Finally, the stained sections were mounted in neutral resin for long-term preservation and microscopic examination. Representative sections with intact articular surfaces were selected for imaging and analysis.

### 4.3. Acquisition of Primary Osteoblasts

At each time point, rats were euthanized with sodium pentobarbital. After euthanasia, the animals were immersed in 75% ethanol for 3–5 min for disinfection. The quadriceps and gastrocnemius muscles were carefully dissected, exposing the femur and tibia after removal of the fibula. The knee joint capsule was incised anteriorly, and the surrounding ligaments were cut with ophthalmic scissors to allow separation of the femur and tibia.

To obtain osteoblasts, the articular cartilage was carefully removed, and the subchondral bone was dissected into 0.5 mm × 0.5 mm fragments. These bone pieces were placed in D-Hanks buffer (Solarbio, Beijing, China) supplemented with penicillin (3 × 10^5^ U/L) and streptomycin (3 × 10^5^ U/L) and washed three times.

A two-step enzymatic digestion was performed to isolate primary osteoblasts: digestion with 0.25% trypsin at 37 °C for 10 min, followed by removal of the trypsin solution, and digestion with a mixture of collagenase type I (5 mg) and hyaluronidase (2.5 mg) in Dulbecco’s modified Eagle’s medium (DMEM, Corning, NY, USA) at 37 °C for 90 min with gentle shaking every 5 min.

The digestion was stopped with the serum-containing medium, and the suspension was centrifuged at 1000 rpm for 6 min to collect the cell pellet. Osteoblasts were resuspended in the culture medium and incubated at 37 °C in a 5% CO_2_ atmosphere with saturated humidity. Cells were purified by differential adhesion and passaged at confluence.

### 4.4. Cell Culture

Primary osteoblasts from the ACLT and sham groups were cultured in Dulbecco’s modified Eagle’s/F12 medium (DMEM/F12, Corning, NY, USA) supplemented with 10% fetal bovine serum (FBS, Corning, NY, USA), 100 U/mL penicillin, and 100 μg/mL streptomycin. Cells were maintained at 37 °C in a 5% CO_2_ incubator, and the medium was changed every three days. Osteoblasts were harvested at the indicated time points for metabolomic analysis.

### 4.5. Identification of Primary Osteoblasts via Morphological Observation and Alkaline Phosphatase (ALP) Staining

To verify the identity of isolated primary cells as osteoblasts, we employed a dual identification strategy combining morphological observation and alkaline phosphatase staining, referencing the classic methodology reported by Wennberg et al. [54]. As a pivotal marker of early osteoblast differentiation, ALP activity is closely associated with osteoblast functional status, making ALP staining an indispensable approach for osteoblast identification.

Isolation and culture of primary osteoblasts were performed meticulously according to the methods described above. After seeding into 6-well plates, dynamic morphological observation was performed immediately after complete cell attachment using an inverted phase-contrast microscope (Olympus IX73, Olympus, Tokyo, Japan). Cell growth patterns, morphological alterations, and characteristic structural features were systematically documented.

The ALP staining procedure was performed as follows: First, adherent cells were gently rinsed three times with phosphate-buffered saline (PBS, Solarbio, Beijing, China) to effectively remove residual culture medium and metabolic byproducts, thereby preventing potential interference with subsequent staining. The cells were then fixed with 4% paraformaldehyde (PFA) at room temperature for 10–15 min to stabilize cellular structures and protein components. After fixation, three additional PBS rinses were performed to thoroughly remove residual fixative. ALP staining solution (Sigma-Aldrich, St. Louis, MO, USA) was then added, and the 6-well plates were incubated at 37 °C in the dark. Under optimal reaction conditions, ALP catalyzes the hydrolysis of substrates in the staining solution, producing insoluble blue-violet precipitates that visually reflect the intracellular distribution of ALP activity. Once clear staining was achieved, high-resolution images of the cells were captured using a stereomicroscope (Leica M205FA, Leica Microsystems, Wetzlar, Germany). By analyzing the distribution density and staining intensity of the blue-violet precipitates, the ALP activity level was comprehensively evaluated, confirming the osteoblastic characteristics of the cells.

### 4.6. Extraction of Intracellular Metabolites

Intracellular metabolites were extracted using a two-phase solvent extraction method [55]. The culture medium was discarded, and the cells were washed three times with pre-cooled PBS (Solarbio, Beijing, China) at 4 °C to remove residual medium. An amount of 3 mL of pre-cooled methanol (−80 °C) was added to stop metabolic activity, and the cells were scraped into a 15 mL centrifuge tube.

A mixture of methanol, chloroform, and water (4:4:2.85, *v*/*v*) was used for extraction. The aqueous phase was collected and lyophilized in a lyophilizer freezer to obtain intracellular metabolites. The lyophilized metabolite powder was dissolved in 550 μL of phosphate buffer (50 mM PO_4_^3−^, 0.05 mM TSP, pH 7.4) and then transferred into a 5 mm NMR tube for subsequent NMR experiments.

Each NMR sample corresponded to osteoblasts isolated from a single rat (*n* = 8 per subgroup at each time point). To ensure biological reproducibility, independent biological replicates (cells from different rats) were analyzed, rather than technical replicates of the same sample. This approach is consistent with standard practice in metabolomic studies where biological variability is prioritized to capture inter-individual metabolic differences [55,56].

### 4.7. Acquisition of NMR Spectra

One-dimensional (1D) ^1^H-NMR spectra were acquired at 298 K on a Bruker Avance III 850 MHz NMR spectrometer, using the NOESYGPPR1D pulse sequence [RD-G1-90°-τ-90°-τ_m_-G2-90°-ACQ]. The relaxation delay (RD) was set to 2 s, the short delay (τ) was set to 4 μs, and the mixing time (τ_m_) was set to 10 ms. A spectral width of 20 ppm was used, with a total of 128 transients collected into 64k data points. The acquisition time was 1.93 s, with an additional relaxation delay of 4 s, and 64 scans were performed.

Two-dimensional (2D) ^1^H-^1^H total correlation spectroscopy (TOCSY) spectra were recorded with a spectral width of 10 ppm in both dimensions, using a data matrix of 2048 × 256 points and a relaxation delay time (RD) of 1.5 s. Two-dimensional ^1^H-^13^C heteronuclear single quantum coherence (HSQC) spectra have a spectral width of 10 ppm in the ^1^H direction and 110 ppm in the ^13^C direction, using a data matrix of 1024 × 128 points.

### 4.8. Processing of NMR Spectra

All 1D NMR spectra were processed using MestReNova (v9.0) for phase and baseline correction and chemical shift calibration, referenced to the methyl peak of TSP. To eliminate bias, the water resonance region (4.70–4.85 ppm) was excluded.

Spectra from 0.50–8.75 ppm were segmented into 0.001 ppm bins and integrated. Non-overlapping resonances were carefully selected for quantification, and peak integrals were normalized to the total spectral sum to ensure accurate relative metabolite concentration.

To minimize variability due to animal age, metabolite concentrations in the ACLT group were normalized to their counterparts in the sham group (ACLT/sham ratios) to provide relative metabolite concentrations and reduce potential confounding effects. This normalization strategy not only accounts for baseline metabolic differences but also ensures that observed changes are specifically due to ACL injury rather than systemic surgical stress. 

### 4.9. Resonance Assigments of Metabolites

The 1D ^1^H-NMR spectra were analyzed using Chenomx NMR Suite (v8.5, Chenomx Inc., Edmonton, AB, Canada) for metabolite peak fitting and resonance assignment. The Human Metabolome Database (HMDB v5.0, http://www.hmdb.ca/ (accessed on 25 December 2024)) was selected as the primary reference based on its comprehensive coverage of >220,000 metabolite entries with experimentally determined NMR chemical shifts from controlled pH (7.0 ± 0.2) and temperature (298 K) conditions, as documented in its technical specifications [57]. To account for inherent chemical shift variability between experimental settings, compensation was applied using the pH-dependent δ-correction factors established by Bhinderwala et al. [58]. Structural assignments were further verified using a graph-theoretic approach [59] that reconstructs molecular connectivity networks from 2D NMR correlations, thereby providing quantum chemically topological constraints.

To resolve structural ambiguities inherent in 1D NMR analyses, orthogonal validation was conducted using 2D ^1^H-^1^H TOCSY for establishing through-space spin system connectivity, complemented by ^1^H-^13^C HSQC spectroscopy to unambiguously resolve carbon-proton coupling networks. This orthogonal validation strategy is consistent with the level 2 confidence criteria defined by the Metabolomics Standards Initiative (MSI), which requires the agreement of two orthogonal analytical properties (e.g., retention time/index and spectral match) or the use of two independent spectroscopic techniques for the identification of putative compounds [56].

### 4.10. Multivariate and Univariate Statistical Analyses of NMR Dataset

The NMR dataset of osteoblast extracts was analyzed using SIMCA-P (v12.0.1). Normalized spectral data were Pareto scaled, followed by unsupervised principal component analysis (PCA) and supervised orthogonal partial least squares discriminant analysis (OPLS-DA) to assess group trends and metabolic variations. The robustness of the OPLS-DA model was assessed using a 200-fold cross-validation response permutation test.

Intracellular concentrations of metabolites at different time points post-ACLT were compared using one-way ANOVA, with results expressed as mean ± SD. A *p*-value < 0.05 was considered statistically significant. In the OPLS-DA model, metabolites with variable importance in projection (VIP) > 1 and *p* < 0.05 were classified as characteristic metabolites.

### 4.11. Metabolic Pathway Analysis

Metabolic pathway analysis was performed based on relative metabolite concentrations using the MetaboAnalyst web server (version 5.0, https://www.metaboanalyst.ca/ (accessed on 30 December 2024)). Significantly altered pathways over the time course after ACLT were identified using a threshold of *p* < 0.05 and pathway impact value (PIV) > 0.2.

### 4.12. Data Analysis

One-way ANOVA (GraphPad prism v8.0.2) was performed for statistical analysis of the experimental results, followed by post hoc analysis to assess significant effects where applicable. *p*-values were corrected using the false discovery rate (FDR) method with a threshold of 5%. To preserve the integrity of the original data, all results were retained without exclusion, maintaining the characteristics of the raw dataset. Statistical significance was defined as follows: ns, adjusted *p* > 0.05; *, adjusted *p* < 0.05; **, adjusted *p* < 0.01; ***, adjusted *p* < 0.001; ***, adjusted *p* < 0.0001.

## 5. Conclusions

Our study demonstrates that metabolic changes in osteoblasts following anterior cruciate ligament (ACL) injury are closely linked to the pathogenesis of osteoarthritis (OA). Significant perturbations in energy metabolism, particularly in the glucose and branched-chain amino acid (BCAA) pathways, suggest promising therapeutic targets for mitigating OA progression. In addition, the observed fluctuations in alanine metabolism underscore the complexity of osteoblast energy regulation in response to joint injury.

Future research should prioritize validating these findings in human OA tissues and exploring combinatorial therapies targeting both metabolic and inflammatory pathways. Future research should focus on elucidating the mechanistic pathways driving these metabolic shifts and exploring targeted metabolic interventions as potential therapeutic strategies for OA management.

## Figures and Tables

**Table 1:** Quantitative comparison of metabolite concentrations in osteoblasts at different time points after ACLT.

Metabolite	Mean ± SD				One-Way ANOVA				
	0 W	4 W	8 W	12 W	4 W vs. 0 W	8 W vs. 4 W	12 W vs. 8 W	F	P
Leucine	0.948 ± 0.061	0.979 ± 0.012	1.014 ± 0.036	1.259 ± 0.079	ns	ns	****↑	56.538	<0.0001
Isoleucine	0.940 ± 0.064	1.031 ± 0.014	1.021 ± 0.048	1.245 ± 0.043	***↑	ns	****↑	64.255	<0.0001
Valine	0.973 ± 0.060	1.015 ± 0.018	0.951 ± 0.044	1.330 ± 0.052	ns	*↓	****↑	116.284	<0.0001
Alanine	1.000 ± 0.044	0.829 ± 0.015	0.776 ± 0.050	1.378 ± 0.130	****↓	ns	****↑	110.373	<0.0001
Acetate	0.631 ± 0.252	3.702 ± 0.219	0.866 ± 0.296	0.588 ± 0.043	****↑	****↓	*↓	363.034	<0.0001
Glutamate	1.086 ± 0.314	0.340 ± 0.071	1.298 ± 0.075	0.912 ± 0.107	****↓	****↑	***↓	44.734	<0.0001
Pyroglutamate	1.113 ± 0.061	1.509 ± 0.004	0.897 ± 0.067	0.501 ± 0.072	****↑	****↓	****↓	419.480	<0.0001
Dimethylamine	1.230 ± 0.268	0.303 ± 0.031	1.395 ± 0.172	2.418 ± 0.575	****↓	****↑	****↑	55.457	<0.0001
Aspartate	0.810 ± 0.106	0.179 ± 0.040	0.750 ± 0.036	0.899 ± 0.008	****↓	****↑	****↑	240.328	<0.0001
Glutathione	1.276 ± 0.032	0.382 ± 0.007	1.539 ± 0.307	1.344 ± 0.244	****↓	****↑	ns	54.639	<0.0001
DMF	0.885 ± 0.140	0.409 ± 0.071	1.213 ± 0.183	1.165 ± 0.363	***↓	****↑	ns	22.968	<0.0001
β-alanine	0.910 ± 0.065	0.366 ± 0.051	1.348 ± 0.254	1.068 ± 0.221	****↓	****↑	**↓	45.568	<0.0001
Taurine	0.963 ± 0.121	0.982 ± 0.051	0.827 ± 0.107	0.865 ± 0.101	ns	**↓	ns	4.608	<0.01
Glycine	0.994 ± 0.493	0.212 ± 0.114	2.220 ± 0.436	0.921 ± 0.115	****↓	****↑	****↓	48.372	<0.0001
Threonine	1.047 ± 0.043	0.416 ± 0.009	1.506 ± 0.108	1.061 ± 0.011	****↓	****↑	****↓	470.833	<0.0001
Creatine	1.056 ± 0.075	0.315 ± 0.004	1.558 ± 0.279	1.362 ± 0.250	****↓	****↑	*↓	65.303	<0.0001
Creatine phosphate	1.502 ± 0.003	0.272 ± 0.017	1.134 ± 0.081	1.519 ± 0.419	****↓	****↑	**↑	59.916	<0.0001
Myo-inositol	1.167 ± 0.109	0.445 ± 0.022	1.529 ± 0.136	0.826 ± 0.066	****↓	****↑	****↓	196.117	<0.0001
Lactate	1.116 ± 0.119	5.450 ± 0.375	0.713 ± 0.107	1.135 ± 0.227	****↑	****↓	**↑	736.619	<0.0001
Glucose	0.976 ± 0.081	5.847 ± 0.430	0.814 ± 0.200	1.454 ± 0.611	****↑	****↓	**↑	304.263	<0.0001
Tyrosine	1.025 ± 0.033	1.067 ± 0.036	1.160 ± 0.136	1.488 ± 0.104	ns	*↑	****↑	44.589	<0.0001
Histidine	1.158 ± 0.402	0.967 ± 0.367	1.131 ± 0.127	1.453 ± 0.162	ns	ns	*↑	3.867	0.020
Imidazole	0.898 ± 0.352	0.029 ± 0.014	0.960 ± 0.446	0.047 ± 0.034	****↓	****↑	****↓	26.218	<0.0001
Phenylalanine	1.009 ± 0.050	0.921 ± 0.030	1.110 ± 0.123	1.296 ± 0.073	*↓	****↑	****↑	34.683	<0.0001
NADP^+^	1.767 ± 0.293	0.295 ± 0.056	0.954 ± 0.107	1.045 ± 0.103	****↓	****↑	ns	104.224	<0.0001
Formate	1.104 ± 0.042	0.613 ± 0.022	1.077 ± 0.051	1.189 ± 0.062	****↓	****↑	****↑	244.659	<0.0001

Note: Data are expressed as mean ± SD. Statistical significance was determined by one-way ANOVA with pairwise comparisons between 4 W vs. 0 W, 8 W vs. 4 W and 12 W vs. 8 W. Levels of significance were determined following the correction for multiple comparisons by controlling the false discovery rate (FDR), with the desired false discovery rate set at 0.05. Given this FDR-controlled analysis, the traditional asterisk symbols are used to denote significance based on the FDR-adjusted *p*-values, as follows: ns (not significant), * for adjusted *p* < 0.05, ** for adjusted *p* < 0.01, *** for adjusted *p* < 0.001, and **** for adjusted *p* < 0.0001. ↑ (increase), ↓ (decrease); Red and blue indicate increased and decreased metabolite concentrations, respectively, compared to the previous time point.

**Table 2:** Characteristic metabolites identified from OPLS-DA analysis of osteoblasts at different time points post-ACLT.

Metabolite	4 W vs. 0 W		8 W vs. 4 W		12 W vs. 8 W	
	VIP	Significance	VIP	Significance	VIP	Significance
Glucose	**2.297**	****↑	**2.190**	****↓	1.311	**↑
Lactate	**2.167**	****↑	**2.122**	****↓	0.949	**↑
Acetate	**1.820**	****↑	**1.638**	****↓	0.690	*↓
NADP^+^	**1.251**	****↓	0.788	****↓	0.440	ns
Creatine phosphate	**1.151**	****↓	0.904	****↓	0.922	**↑
Glycine	0.886	****↓	**1.358**	****↑	**1.839**	****↓
Creatine	0.891	****↓	**1.073**	****↑	0.716	*↓
Glutathione	0.980	****↓	**1.026**	****↑	0.730	ns
Threonine	0.824	****↓	**1.016**	****↑	**1.094**	****↓
Dimethylamine	0.988	****↓	**1.014**	****↑	**1.550**	****↑
Myo-inositol	0.880	****↓	**1.012**	****↑	**1.377**	****↓
Imidazole	0.931	****↓	0.875	****↑	**1.505**	****↓
Alanine	0.421	****↓	0.178	ns	**1.260**	****↑
Valine	0.207	ns	0.237	*↓	**1.012**	****↑
Pyroglutamate	0.648	****↑	0.757	****↓	**1.009**	****↓

Note: Characteristic metabolites were identified using the criteria VIP ≥ 1 and adjusted *p* < 0.05 for pairwise comparisons between 4 W vs. 0 W, 8 W vs. 4 W, and 12 W vs. 8 W. VIP scores and adjusted *p*-values are reported for each metabolite at each time point. Statistically significant changes in metabolite concentrations are indicated as follows: ↑ (increase), ↓ (decrease); ns (not significant, adjusted *p* > 0.05), * (adjusted *p* < 0.05), ** (adjusted *p* < 0.01), **** (adjusted *p* < 0.0001). Metabolites with VIP ≥ 1 and adjusted *p* < 0.05 are highlighted in bold.

**Table 3:** Significantly altered metabolic pathways in osteoblasts over the time course after ACLT.

No.	Metabolic Pathway	4 W vs. 0 W	8 W vs. 4 W	12 W vs. 8 W
1	Glutathione metabolism	√	√	√
2	Histidine metabolism	√	√	√
3	Alanine, aspartate, and glutamate metabolism	√	√	√
4	Glycine, serine, and threonine metabolism	√	√	√
5	Phenylalanine metabolism	√	√	√
6	D-glutamine and D-glutamate metabolism	√	√	√
7	Phenylalanine, tyrosine, and tryptophan biosynthesis	√	√	√
8	Taurine and hypotaurine metabolism		√	

Note: Metabolic pathway analysis was performed for pairwise comparisons between 4 W vs. 0 W, 8 W vs. 4 W, and 12 W vs. 8 W. A tick (√) indicates pathways that showed significant alterations at the respective time points.

## Data Availability

The raw data supporting the conclusions of this article will be made available by the authors on request.

## Supplementary Materials

The following supporting information can be downloaded at https://www.mdpi.com/article/10.3390/molecules30112255/s1. Figure S1: Microscopic observation and ALP staining results of primary osteoblasts; Figure S2: Representative 2D ^1^H-^1^H TOCSY spectrum of aqueous metabolites extracted from osteoblasts; Figure S3: Representative 2D ^1^H-^13^C HSQC spectrum of aqueous metabolites extracted from osteoblasts; Figure S4: Comparison of metabolic profiles of osteoblasts at the 0 W and 8 W time points after ACLT; Figure S5: Key metabolic pathways associated with the onset and progression of OA after ACLT; Table S1: Resonance assignments of aqueous metabolites derived from osteoblasts based on 1H-NMR spectra.
