# Supplementary material for: Metabolomic Profiling of Osteoblasts in Rat Subchondral Bone Following Anterior Cruciate Ligament Injury
Source: Molecules. 2025 May 22;30(11):2255. doi: 10.3390/molecules30112255 (PMC12156015; doi:10.3390/molecules30112255)
Supplement: Supplementary file 1 [file molecules-30-02255-s001.zip › molecules-3549280-supplementary.pdf]

Supplementary Materials

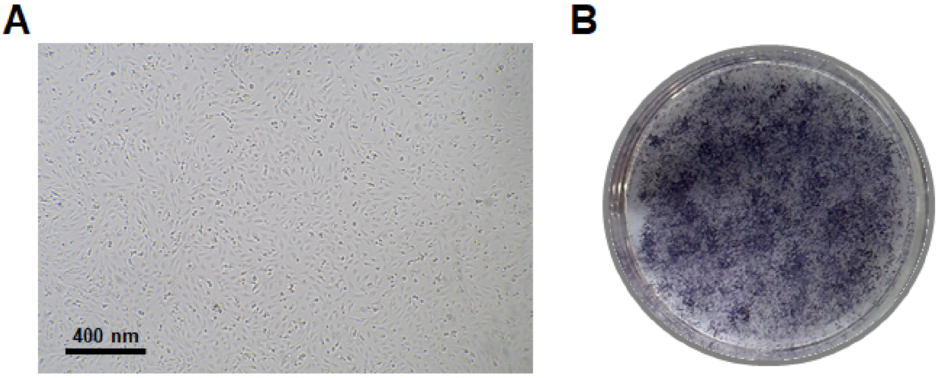

Figure S1. Microscopic observation and ALP staining results of primary osteoblasts. (A) Microscopic view of primary osteoblasts. The cells were observed under a microscope, showing characteristic morphological features. Scale bar: 400 nm. (B) Alkaline phosphatase (ALP) staining of primary osteoblasts. The blue-violet precipitate generated by ALP staining indicates the presence of ALP activity in the cells, confirming the osteogenic nature of the isolated primary osteoblasts.

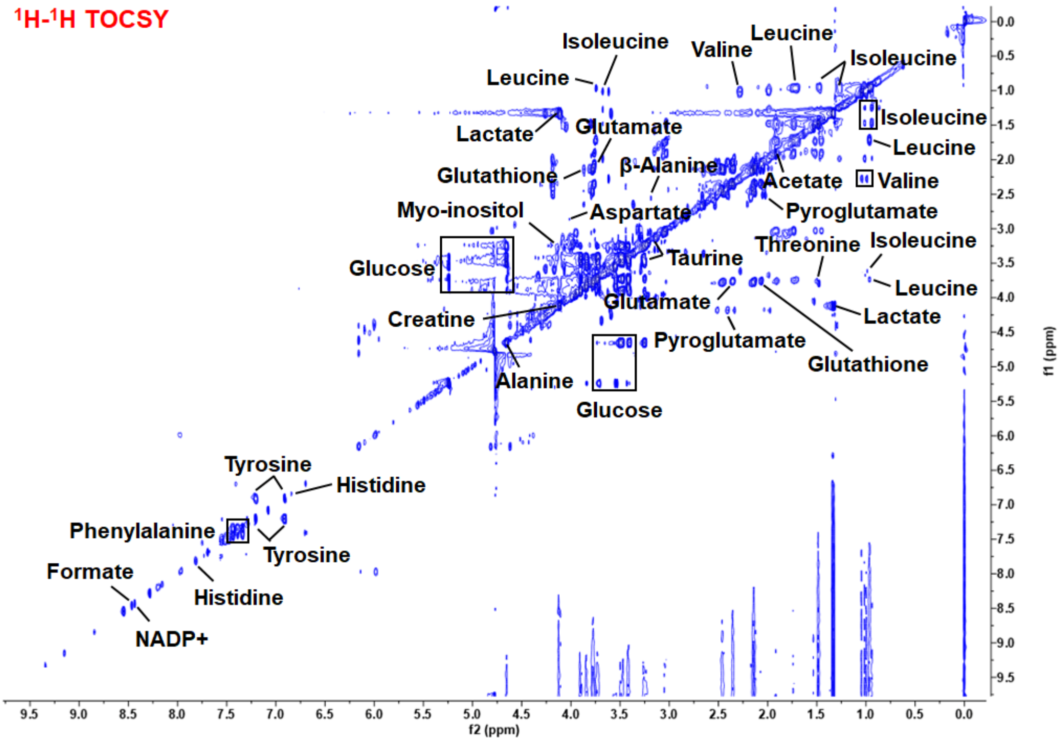

Figure S2. Representative 2D ^{1}H-^{1}H TOCSY spectrum of aqueous metabolites extracted from osteoblasts. The spectrum was recorded using a Bruker Avance III 850 MHz NMR spectrometer at 298 K and pH 7.4.

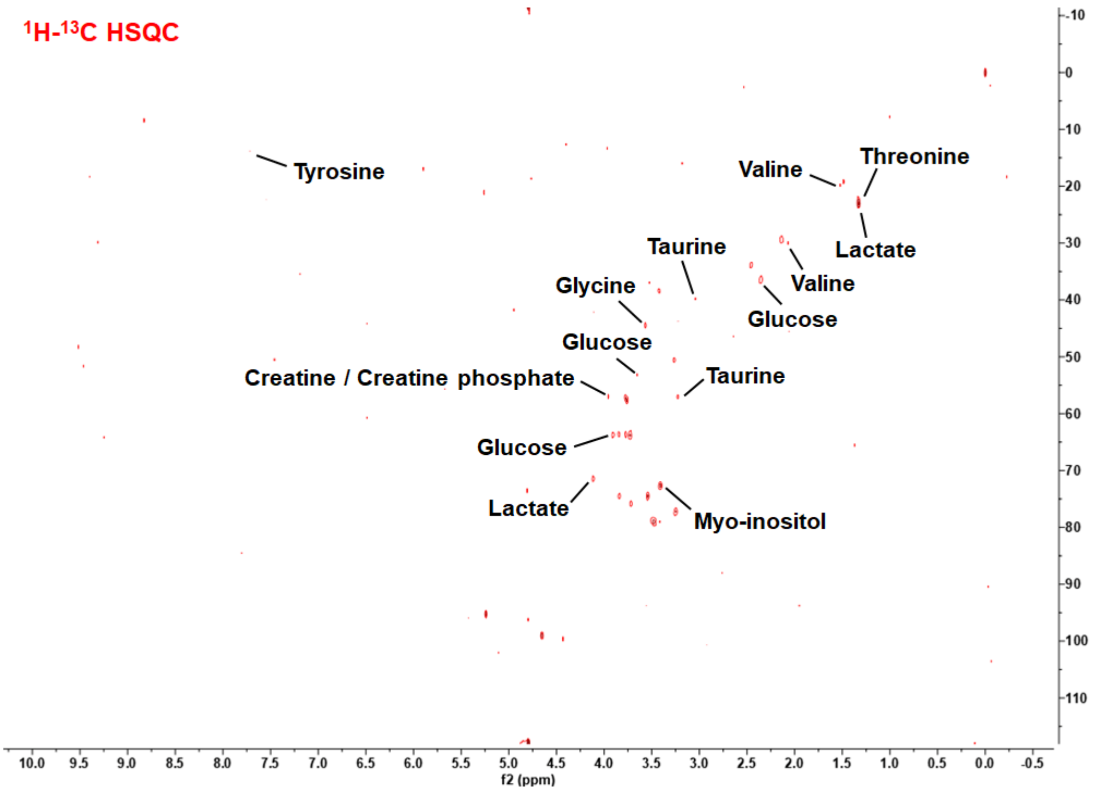

Figure S3. Representative 2D ^{1}H-^{13}C HSQC spectrum of aqueous metabolites extracted from osteoblasts. The spectrum was recorded using a Bruker Avance III 850 MHz NMR spectrometer at 298 K and pH 7.4.

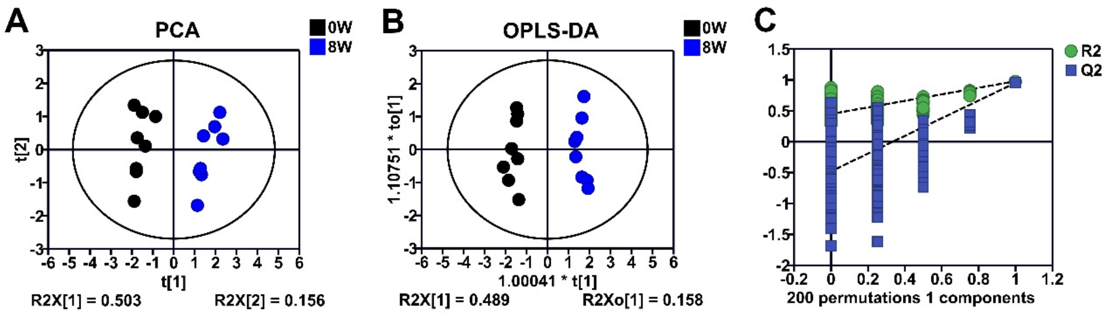

Figure S4. Comparison of metabolic profiles of osteoblasts at the 0W and 8W time points after ACLT. (A) PCA score plot. (B) OPLS-DA score plot. (C) Cross-validation plot illustrating the good robustness of the OPLS-DA model using a randomized permutation test (n = 200).

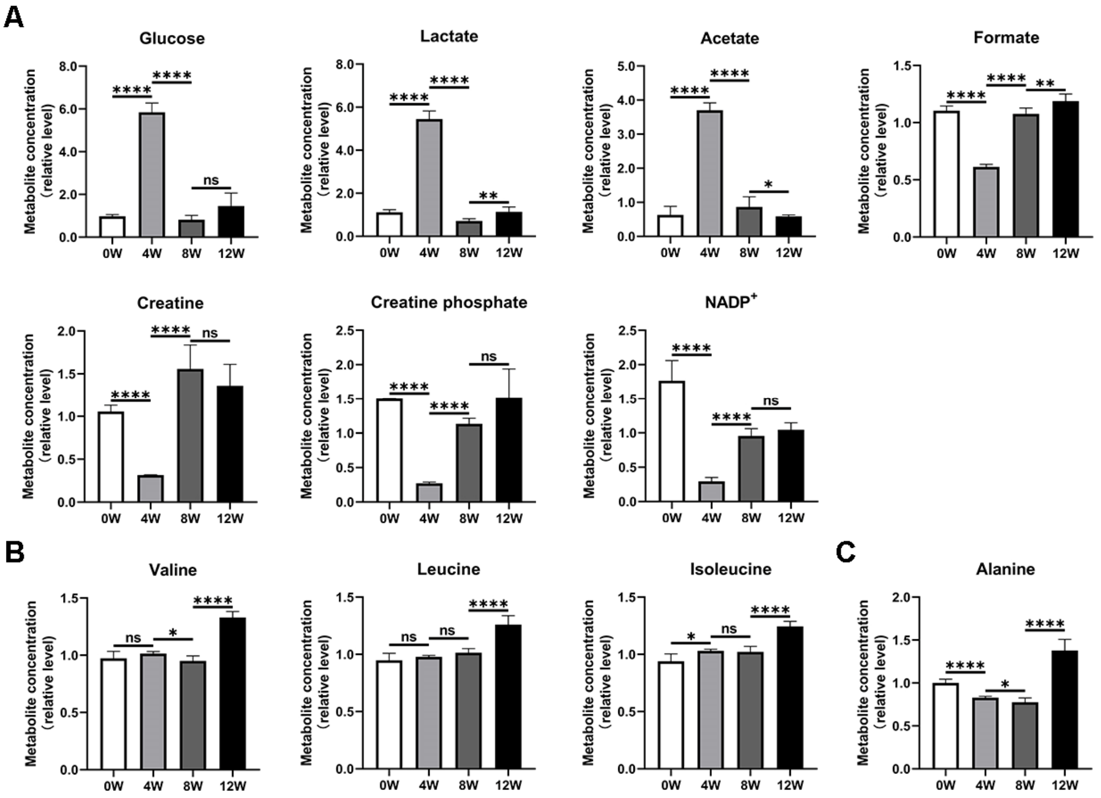

Figure S5. Key metabolic pathways associated with the onset and progression of OA after ACLT. This figure illustrates metabolic changes in three critical pathways involved in OA pathogenesis: (A) energy substrate metabolism, (B) branched-chain amino acid (BCAA) metabolism, and (C) alanine metabolism. Statistical significance was assessed by one-way ANOVA, with significance levels indicated as: ns (not significant, adjusted-$p > 0.05$), * adjusted-$p < 0.05$, ** adjusted-$p < 0.01$, **** adjusted-$p < 0.0001$.

Table S1. Resonance assignments of aqueous metabolites derived from osteoblasts based on ^{1}H-NMR spectra.

No.	Metabolite	δ^1H (ppm) and Resonance Assignment
1	Leucine	0.960 (t, δ-CH_3), 0.970 (d, γ-CH_3)
2	Isoleucine	0.940 (t, δ-CH_3), 1.015 (d, γ-CH_3)
3	Valine	0.985 (d, γ-CH_3), 1.047 (d, β-CH_3)
4	Alanine	1.478 (d, β-CH_3)
5	Acetate	1.920 (s, CH_3)
6	Glutamate	2.065 (m, β-CH_2), 2.352 (m, γ-CH_2)
7	Pyroglutamate	2.412 (m, C3-H), 2.517 (m, C4-H), 4.182 (m, C5-H)
8	Dimethylamine	2.725 (s, N-CH_3)
9	Aspartate	2.683 (dd, β-CH_2), 2.817(dd, α-CH)
10	Glutathione	2.515 (m, Cys-β-CH_2), 2.575 (m, Glu-γ-CH_2), 2.975 (m, Gly-α-CH_2)
11	DMF	3.015 (s, N(CH_3)2)
12	β-Alanine	3.185 (t, β-CH_2)
13	Taurine	3.265 (t, -SO_3-CH_2), 3.420 (t, -NH-CH_2)
14	Glycine	3.552 (s, α-CH_2)
15	Threonine	1.330 (d, γ-CH_3), 3.595 (d, β-CH)
16	Creatine	3.040 (s, N-CH_3), 3.933 (s, N-CH_2)
17	Creatine phosphate	3.045 (s, N-CH_3), 3.965 (s, PO4-CH_2)
18	Myo-inositol	3.267 (t, H5), 3.540 (dd, H4), 3.625 (t, H6), 4.065 (t, H2)
19	Lactate	1.332 (d, CH_3), 4.113 (q, CH)
20	Glucose	3.248 (dd, H2), 3.412 (q, H4), 3.467 (dd, H3), 3.494 (t, H5), 3.540 (dd, H1), 3.717 (t, H6), 3.726 (dd, H6'), 3.767 (m, H4'), 3.900 (dd, H6"), 4.651 (d, H1α), 5.238 (d, H1β)
21	Tyrosine	6.900 (d, H3/H5), 7.195 (d, H2/H6)
22	Histidine	7.065 (s, H2), 7.800 (s, H4)

23	Imidazole	7.280 (d, H2)
24	Phenylalanine	7.330 (d, H2/H6), 7.380 (t, H3/H5), 7.430 (t, H4)
25	$NADP^+$	6.045 (d, Nicotinamide-H5), 6.095 (d, Nicotinamide-H6), 8.175 (s, Adenine-H2), 8.200 (m, Ribose-H1'), 8.430 (s, Adenine-H8), 8.845 (d, Phosphate-H), 9.150 (d, Phosphate-H'), 9.340 (s, Ribose-H2')
26	Formate	8.460 (s, H)
27	AXP	6.15 (d, ribose-H1'), 8.10 (s, adenine-H8)
28	IMP	6.15 (d, H1'), 8.10 (s, hypoxanthine-H8)

Note: Multiplicity: s, singlet; d, double; t, triplet; q, quartet; m, multiple; dd, double of double. AXP: Unresolved ATP/ADP/AMP mixture; IMP: Tentative assignment (requires 2D NMR confirmation).
